# Diphtheria

**Published:** 1886-01

**Authors:** 


### DIPHTHERIA.

Dr. Nagle, of the Bureau of Vital Statistics, declares diphtheria to be rapidly on the increase in the city notwithstanding the many "absolute cures" that have been tried in various places and at different times. It is, says the Doctor, "a dangerous disease, and if it once gets beyond control no one can tell where its ravages will end. Prompt action should be taken by every one where it exists to keep it within bounds. The health officials are doing all they can to keep the upper hand of the dread disease. Two men are disinfecting houses, utensils and bedding where danger is serious. While diphtheria is not always so malignant as small-pox, it is fully as difficult to control when once it gets started in a large city like this. The law should be strictly enforced and no one allowed to attend the funerals of persons having died of diphtheria. The disease is just as fatal as ever it was and of the cases reported to us 50 per cent. of the patients die. Not nearly all those who have diphtheria in the city are ever heard of at the Board of Health unless they die. Then, of course, applications for burial permits are sent in."

The Doctor exhibited to the reporter a book and read from it the following, which, he stated, should be known to everybody:

"Diphtheria is an acute infectious disease in which there is a tendency to the formation of false membrane on mucous and abraded surfaces, accompanied by considerable constitutional disturbances.

"Symptoms :—After an incubation period from twenty-four hours to ten days, shivering and vomiting set in and the temperature increases, the throat is sore with some stiffness about the neck. The fauces become of a dark red, color, the tonsils swoolen, and at the end of two days from the beginning of the disease, a quantity of minute white points appear on the surfaceof both sides of the fauses.

"As these specks increase in numbers they coalesce and form a thick yellowish white membrane. This sometimes consists of a single piece, but is often scattered over the surface in separate patches. The cervical glands become enlarged, the tongue coated with white or brown fur, the pulse temperature increased and the urine albuminous.

"In the next stages the membrane separates after leaving unhealthy sloughing ulcers.

"In this stage recovery may take place, or death may result from exhaustion. During any period of the illness asphyxia, resulting from the production of false membrane in the larnyx or bronchial tubes, may be fatal. In the course of the disease a roseolous rash may appear over the body.

"Convalescence after diphtheria is very slow. Often paralysis of the groups of muscles shows itself within six months from the beginning of the attack. The removal of the false membrane is of no avail.

"The difference between diphtheria and scarletina is that in diphtheria the throat is of a deep red color, with patches of thick yellow membrane, in scarlet fever there are eruptions, and the bright red is uniformly distributed and the tonsils equally swollen."

Dr. T. D. Swift, another authority on diphtheria, thought there was no cause for alarm, although diphtheria is on the increase and is worse than it has been in two or three years. He was attending to patients in the DeWitt Dispensary, Southern District, on the east side, lying between Fourteenth and Forty-eighth streets. He did not think the situation alarming.

A learned physician said he had spent years in studying the disease and was called by the foolish public "a distinguished authority" on diphtheria, yet in spite of all his knowledge—in spite of all his successes in curing a great many cases—his child had died of the dreadful disease. "It baffles the best of us at times, Often when there is good drainage, good water, good ventilation, plenty of good food and proper exercise the children are swept away. The cause may be remote—so trivial as to be overlooked even by the careful eye of the most experienced physician."

Dr. C. E. Billington, also of the DeWitt Dispensary, and the author of valuable data in the *Medical Record* on diphtheria, said last evening that it was very difficult to tell whether diphtheria was caused by derangement of the blood and the system or whether it was caused by local troubles in the throat. The profession was divided. Some physicians thought the diseased throat affects the entire system, while others were of the opinion that the bad condition of the blood and body caused the throat to inflame and a membrane to form in the breathing passage.

Dr. Nagle stated that the first case of diphtheria occurred in 1857. At that time the disease was called malignant sore throat. The record since then has been as follows :

| *Year.* | *No. of Deaths.* | *Date.* | *No. of Deaths.* |
|---|---|---|---|
| 1860 | 422 | 1873 | 1,151 |
| 1861 | 453 | 1874 | 1,665 |
| 1862 | 594 | 1875 | 2,329 |
| 1863 | 980 | 1876 | 1,750 |
| 1864 | 791 | 1777 | 951 |
| 1865 | 534 | 1878 | 1,007 |
| 1866 | 435 | 1879 | 671 |
| 1867 | 278 | 1880 | 1,390 |
| 1868 | 277 | 1881 | 2,249 |
| 1869 | 328 | 1882 | 1,525 |
| 1870 | 308 | 1883 | 1,009 |
| 1871 | 238 | 1884 | 1,090 |
| 1872 | 446 | | |

The record for the last five months for this year, 1885, is as follows :

| *Date.* | *No. of Cases.* | *No. of Deaths.* |
|---|---|---|
| Aug. 8 | 24 | 9 |
| Aug. 15 | 30 | 17 |
| Aug. 22 | 27 | 13 |
| Aug. 29 | 25 | 14 |
| Sept. 5 | 28 | 18 |
| Sept. 12 | 35 | 14 |
| Sept. 19 | 44 | 23 |
| Sept. 26 | 42 | 28 |
| Oct. 3 | 32 | 11 |
| Oct. 10 | 46 | 17 |
| Oct. 17 | 52 | 24 |
| Oct. 24 | 46 | 19 |
| Oct. 31 | 41 | 21 |
| Nov. 7 | 63 | 27 |
| Nov. 14 | 66 | 26 |
| Nov. 21 | 70 | 25 |
| Nov. 28 | 62 | 25 |
| Dec. 5 | 95 | 42 |
| Dec. 12 | 87 | 44 |

The deaths for the last six weeks in 1884 were 206, for the six weeks just expired, the number is 192.

Deaths from diphtheria per 100,000 inhabitants in the principal cities of the world :

| | | | |
|---|---|---|---|
| Amsterdam | 265 | Paris | 85 |
| Berlin | 245 | Hamburg | 76 |
| Madrid | 225 | Naples | 74 |
| Dresden | 184 | Lisbon | 74 |
| Warsaw | 267 | Stuttgart | 61 |
| Philadelphia | 163 | Rome | 56 |
| Chicago | 146 | Edinburgh | 50 |
| Turin | 127 | Buda Pesth | 50 |
| St. Petersburg | 121 | The Hague | 45 |
| Bucharest | 118 | Vienna | 44 |
| Berne | 115 | London | 44 |
| Munich | 111 | Christiania | 43 |
| Stockholm | 107 | Copenhagen | 42 |
| Malines | 105 | Suburbs of Brussels | 36 |
| Antwerp | 104 | City of Brussels | 35 |
| New York | 91 | | |

The *Siglo Medico*, from which this extract is taken, considers Brussels a highly favored city. It is certainly so in regard to exemption from diphtheria.

